# Molecular basis of floral petaloidy: insights from androecia of *Canna indica*

**DOI:** 10.1093/aobpla/plu015

**Published:** 2014-03-31

**Authors:** Qian Fu, Huanfang Liu, Ana M. R. Almeida, Yanfeng Kuang, Pu Zou, Jingping Liao

**Affiliations:** 1 Key Laboratory of Plant Resources Conservation and Sustainable Utilization, South China Botanical Garden, Chinese Academy of Sciences, Guangzhou 510650, PR China; 2 Graduate University of the Chinese Academy of Sciences, Beijing 100049, PR China; 3 Department of Plant and Microbial Biology and the University and Jepson Herbaria, University of California, Berkeley, CA 94720, USA

**Keywords:** ABC model, *Canna indica*, floral organ identity, MADS-box gene, phylogenetic analysis, real-time PCR.

## Abstract

Floral organs that take on the characteristics of petals can occur in all whorls of the monocot order Zingiberales. In *Canna indica*, the most ornamental or ‘petaloid’ parts of the flowers are of androecial origin and are considered staminodes. However, the precise nature of these petaloid organs is yet to be determined. In order to gain a better understanding of the genetic basis of androecial identity, a molecular investigation of B- and C-class genes was carried out. Two MADS-box genes *GLOBOSA* (*GLO*) and *AGAMOUS* (*AG*) were isolated from young inflorescences of *C. indica* by 3′ rapid amplification of cDNA ends polymerase chain reaction (3′-RACE PCR). Sequence characterization and phylogenetic analyses show that *CiGLO* and *CiAG* belong to the B- and C-class MADS-box gene family, respectively. *CiAG* is expressed in petaloid staminodes, the labellum, the fertile stamen and carpels. *CiGLO* is expressed in petals, petaloid staminodes, the labellum, the fertile stamen and carpels. Expression patterns in mature tissues of *CiGLO* and *CiAG* suggest that petaloid staminodes and the labellum are of androecial identity, in agreement with their position within the flower and with described *Arabidopsis thaliana* expression patterns. Although B- and C-class genes are important components of androecial determination, their expression patterns are not sufficient to explain the distinct morphology observed in staminodes and the fertile stamen in *C. indica*.

## Abstract (precis)

Floral organs usually take on the characteristics of petals in ginger plants. In *Canna indica*, the most ornamental parts of the flowers are considered to be staminode, which means rudimentary and sterile stamen. However, the precise nature of these petaloid organs is yet to be determined. Two floral organ identity genes GLOBOSA (GLO) and AGAMOUS (AG) are isolated from *Canna indica*. Their expression patterns suggest that petaloid staminodes and labellum are of androecium identity, in agreement with their position within the flower. But the current molecular, morphological and anatomical data, are still not sufficient to explain the distinct morphology observed in staminodes and fertile stamen in *Canna indica*.

## Introduction

A landmark accomplishment in plant developmental biology was the proposition of the ABC model of flower organ identity. This model provides a framework for describing a conserved pattern of gene expressions associated with the specification of floral organs in model species (Bowman *et al.* 1991; Coen and Meyerowitz 1991). Soon after its proposition, other floral identity genes including D- and E-class genes were identified and the ABC model was then extended to an ABCDE model (Angenent *et al.* 1995; Pelaz *et al.* 2000; Ditta *et al.* 2004). ABC model genes, with the exception of *APETALA2* (*AP2*) (Jofuku *et al.* 1994), belong to the MADS-box family of transcription factors. In angiosperms, MADS-box transcription factors are important components of various developmental processes, although the most well-characterized MADS-box genes to date are involved in floral organ identity and initiation (Theißen and Saedler 2001).

The ABC model has been extensively characterized due to molecular studies in two model plants, *Arabidopsis thaliana* (Yanofsky *et al.* 1990; Jack *et al.* 1992; Mandel *et al.* 1992; Goto and Meyerowitz 1994; Gustafsonbrown *et al.* 1994) and *Antirrhinum majus* (Sommer *et al.* 1990; Huijser *et al.* 1992; Trobner *et al.* 1992; Bradley *et al.* 1993). Studies in other angiosperm lineages generally support the idea that the expression patterns described by the ABC model are widely conserved, at least among eudicots. However, the extent to which the eudicot-based ABC model of flower organ identity describes floral morphologies in monocots is still not well understood (Ambrose *et al.* 2000).

The role of A-class genes appears to be conserved only in the lineage containing *Arabidopsis* (Causier *et al.* 2010), and to date the A-function of the ABC model has not been shown to play a role in organ identity in monocots. Studies carried out in maize, rice and wheat (Mena *et al.* 1996; Kang *et al.* 1998; Ambrose *et al.* 2000; Munster *et al.* 2001; Meguro *et al.* 2003), however, do indicate that B- and C-class genes and gene activities are somewhat conserved between grasses and eudicots, despite evidence for gene duplication in various lineages. However, the derived morphology of grass flowers results in inconclusive debates regarding the interpretation of the lemma and palea as equivalent to sepals and petals of eudicots (Bowman 1997; Ambrose *et al.* 2000). Also, studies in other monocot lineages such as the Liliaceae show divergent results from those predicted by the canonical BC model. In this family, flowers have two outer whorls of almost identical petaloid organs called tepals. In order to explain such a derived pattern, a modified BC model has been proposed (Van Tunen *et al.* 1993) and ascertained (Kanno *et al.* 2003). In this modified model, B-class genes are expressed in whorl 1 as well as whorls 2 and 3, resulting in similar expression patterns in whorls 1 and 2 and, therefore, similar petaloid structure (Kanno *et al.* 2007). However, the expression patterns of B-class genes in asparagus, for instance, do not fit this model (Kanno *et al.* 2004).

Furthermore, the intricate morphology and unprecedented diversity of orchid floral morphology has resulted in a proposed ‘orchid code’ that builds on the BC model. This idea assumes that the identity of the different perianth organs is specified by the combinatorial interaction of four *DEF*-like MADS-box genes with other floral homeotic genes (Mondragón-Palomino and Theißen 2008, 2009).

Owing to the lack of consistency regarding the conservation of the BC model in monocots, it is likely that further studies on other non-grass, petaloid monocots will provide a novel understanding of the evolution of floral organ identity specification outside the eudicots.

The Zingiberales is an order of tropical monocots and consists of eight families (Tomlinson 1962; Dahlgren and Rasmussen 1983). The families are often divided into two groups: the paraphyletic ‘banana group’ (including Musaceae, Streliziaceae, Heliconiaceae and Lowiaeae) with five (occasionally six) fertile stamens, and the monophyletic ‘ginger group’ (including Zingiberaceae, Costaceae, Cannaceae and Marantaceae) with one or a half fertile stamen (Kress 1990; Rudall and Bateman 2004). Thus, an impressive reduction in the number of fertile stamens occurred in the ancestor of the ginger clade.

The Cannaceae family is derived within the Zingiberales with only a single fertile stamen, further reduced to a single theca (half of a fertile stamen) with a prominent, expanded petaloid appendage (Kirchoff 1983). For many years, the interpretation of the androecium members of the Cannaceae has sparked much debate, especially regarding the developmental origins of the stamen and the labellum in *Canna indica* (see the brief review by Miao *et al.* 2014). In recent years, this scientific problem has been the subject of both morphological observations and molecular studies. Almeida *et al.* (2013) combined a developmental study of the petaloid fertile stamen with data on the expression of three B-class and two C-class floral organ identity genes to elucidate the organogenesis of the petaloid stamen and staminodes. In that study, the authors (Almeida *et al.* 2013) proposed that the canonical BC model is not sufficient to explain petaloidy in the androecial whorl in *Canna* sp. In addition, based on floral vasculature and ontogeny, Miao *et al.* (2014) suggested that the labellum incorporates two androecial members from both the outer and inner whorls, one diverging from the carpellary dorsal bundle and the other from the parietal bundle. The functional stamen also incorporates two androecial members from both the outer and inner whorls. In order to obtain a better understanding of the identity of the androecium members, we carried out a molecular investigation of *C. indica* B- and C-class genes. Based on our molecular results, we discuss the identity of the androecium members in *C. indica* in the context of the current molecular, morphological and anatomical data.

## Methods

### Plant materials

**Figure 1.**
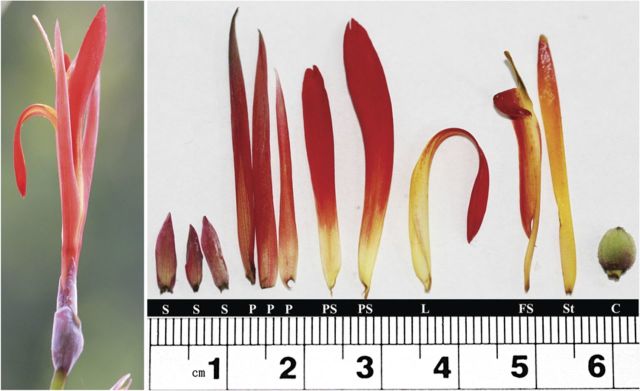
Complete (left) and dissected (right) mature flower of *C. indica*. S, sepal; P, petal; PS, petaloid staminode; L, labellum; FS, fertile stamen; St, style; C, carpel.

*Canna indica* young inflorescences (Fig. 1), with flowers at different developmental stages before anthesis, were collected from living plants growing in the South China Botanical Garden (Guangzhou, Guangdong Province, China). The living materials were collected aperiodically from April to July during the year 2012 as the flower of *C. indica* bloomed.

### RNA extraction and cDNA synthesis

Fresh floral materials were wrapped in aluminium foil, flash frozen in liquid nitrogen and preserved at −80 °C to avoid RNA degradation. To ensure RNase-free materials, the plastic experimental apparatus were dipped in DEPC dH_2_O overnight, whereas all the glass apparatus and mortar are sterilized at 180 °C in the pressure vapour sterilizer overnight before the experiment. Total RNA was extracted from at least 100 mg of floral materials using the Plant RNA Extraction Reagent kit according to the manufacturer's instructions (Bioteke, China). The RNA pellet was re-eluted in 40 μL of RNase-free ddH_2_O and stored at −80 °C. RNA quality was characterized by running 4 μL of extracted RNA through 1.0 % agarose gel electrophoresis. Four microlitres (∼10 μg) of RNA was used in a reverse transcription into cDNA using a poly-T primer designed with software Primer Premier 5 (3′-RACE primer: AAG CAG TGG TAT CAA CGC AGA GTA CTT TTT TTT TTT TTT TTT TTT TTT TTT TTT T) and M-MLV (Invitrogen SuperScriptIII First-Strand Synthesis System for RT-PCR, USA) following the manufacturer's protocol. The success of the reverse transcription reaction was assessed by amplifying 18sRNA from the cDNA, using the following primer pair: 1265S: GGC TAC CAC ATC CAA GGA AG; 1265AS: CCA TTA AAT AGG TAG GAG C.

### Sequence analyses

cDNA was diluted 10 times in dH_2_O, and 4 μL of the dilution was used in 20 μL polymerase chain reaction (PCR) reactions containing 1 μL of 20 μM of each of the forward and reverse primers, 1 U of Taq DNA polymerase (TaKaRa, Japan), 2 μL of dNTP (2 mM) and 2 μL of 10× PCR buffer. The forward primers were designed according to the MADS domain and a RACE-AP Reverse. Two degenerate primer pair combinations were designed according to the conserved region in some monocot plants and were used for each cDNA sample: RACE-AP(R): AAGCAGTGGTATCAACGCAGAGTACGCGGG; GLO-F: ATGGGGCGMGGRAAGATCGAGATCAAG; AG-F: ATGGGSMGRGGRAAGATCGAGATC.

Cycling conditions were set as follows: initial denaturation at 94 °C for 3 min, touchdown procedure from 62 to 48 °C, 15 amplification cycles (94 °C for 30 s, 62 °C for 30 s and 72 °C for 1 min), the cycle of 48 °C followed by another 15 amplification cycles (94 °C for 30 s, 54 °C for 30 s and 72 °C for 1 min) and a final extension step carried out at 72 °C for 10 min.

The full volumes of the PCR reactions were run on 1.0 % agarose gels and bands of the appropriate size (800 bp–1 kb) were excised and cloned on PMD18-T vectors (TaKaRa). Positive colonies were identified by bacterial colony PCR. Then, all the positive clones (six to eight) were picked from each cloning reaction and sequenced using T-vector-specific primers M13-47 and M13-48. All colonies were sequenced to verify identity, and two sequences were submitted to GenBank.

Analyses of the obtained sequences as well as their inferred amino acid translations were performed using the NCBI Blast program (http://www.ncbi.nlm.nih.gov). Alignments of the deduced amino acid sequences were carried out by ClustalX v2.0 (Thompson *et al.* 1997).

Forty MADS-box B-, C- and D-class genes including the two genes of this study were downloaded from NCBI (http://www.ncbi.nlm.nih.gov/nuccore) and a multiple sequence alignment was performed. Gene and species names as well as accession numbers are as follows: *AG* (X53579), *AtSHP1* (M55550), *AtSHP2* (M55553), *AtAGL1* (NM_001084842), *AtAP3* (M86357) and *STK* (U20182) of *A. thaliana*; *AmGLO* (AB516403) and *PLE* (S53900) of *A. majus*; *AhMADS5* (AY621154), *AhMADS6* and *AhMADS8* (AY621156) of *Alpinia hainanensis*; *AoAP3* (EF521817), *AoAG* (DQ286724) and *AoPI* (DQ286723) of *Alpinia oblongifolia*; *FBP11* (X81852), *FBP6* (X68675) and *PMADS2* (X69947) of *Petunia*×*hybrida*; *TAG1* (XM_004232947), *TAGL1* (NM_001247258) and *TAGL11* (NM_001247265) of *Solanum lycopersicum*; *WAG* (AB084577) and *WAG2* (AB465688) of *Triticum aestivum*; *ZMM2* (NM_001111456), *ZmAG* (NM_001112476), *ZMM19* (NM_001111678), *ZMM29* (AJ292961) and *ZMM25* (AJ430638) of *Zea mays*; *BAG1* (M99415) of *Brassica napus*; *LrGLOA* (AB071379) and *LrGLOB* (AB071380) of *Lilium regale*; *TgGLO* (AB094967) of *Tulipa gesneriana*; *OsMADS2* (L37526), *OsMADS3* (L37528), *OsMADS13* (AF151693) and *OsMADS58* (AB232157) of *Oryza sativa*; *NtGLO* (X67959), *NAG1* (L23925) and *GLO* (X67959) of *Nicotiana tabacum*; and *CiAG* (JQ180191) and *CiGLO* (JQ180192) of *C. indica*. The evolutionary history of the genes in the multiple sequence alignment was inferred by using maximum likelihood and the Tamura–Nei model (Tamura and Nei 1993). The Kimura two-parameter model (Kimura 1980) was also tested and resulted in the same tree topology. Initial trees for the heuristic search were obtained automatically by applying neighbour-joining and BioNJ algorithms to a matrix of pairwise distances estimated using the maximum composite likelihood (MCL) approach, and then selecting the topology with superior log likelihood value. Evolutionary analyses were conducted in MEGA6. Bootstrap values on the consensus trees were derived from 1000 bootstrap replicates.

Because the gene sequences (accession numbers GU594899–GU594995) submitted by Bartlett and Specht (2010) and Almeida *et al.* (2013) are only partial CDS, they were not included in this phylogenetic construction.

### Real-time reverse transcription PCR

Four whorls of floral organs were dissected from *C. indica* flowers just before anthesis. The dissected whorls are as follows: sepals in whorl 1, petals in whorl 2, petaloid staminodes and the labellum in whorl 3o (outer androecial whorl), petaloid staminodes and a single fertile stamen in whorl 3i (inner androecial whorl) and carpels in whorl 4. These materials were then separately put into liquid nitrogen to avoid RNA degradation. Meanwhile, a fresh leaf was also collected, flash frozen and used as a reference material. RNA for real-time reverse transcription (RT)-PCR was extracted separately from the four whorls of the floral organs and leaf. RNA extraction was carried out using the Plant RNA Extraction Reagent Kit according to the manufacturer's instructions as described above (Bioteke). The RNA pellet was re-eluted in 40 μL of RNase-free ddH_2_O and stored at −80 °C. RNA quality was tested by running 2 μL of the extracted RNA through a 1.0 % agarose gel electrophoresis (AGE). RNA was quantified using a NanoDrop 1000 spectrophotometer (Thermo Scientific, USA). One microgram of RNA from each sample was reverse transcribed into cDNA for real-time RT-PCR following the manufacturer's protocol (TaKaRa, PrimeScript RT Master Mix Perfect Real Time).

Primer pairs of the reference gene were designed according to the *β-actin* sequence of *Musa acuminata*, a member of the banana family. The primer pairs used for real-time RT-PCR are actin-F: TCCTTTCCCTCTATGCTAGTGGC; actin-R: CCTCCAATCCAGACACTGTACTTA; AG-F: TAGAGATTATGGGGCGAGGAA; AG-R: ATCCGAGTTGGAGGGCAGT; GLO-F: GAGGCACTCCAGATTGGGCTTAC; and GLO-R: TGGGAAAAGGATGACGGGAAG. Reagent concentrations were used as follows: 2× SYBR buffer solution (TaKaRa) 10 μL, RoxII 0.4 μL, dH_2_O 2 μL, reverse primer 0.8 μL (10 μM, 0.052 μg) and forward primer 0.8 μL (10 μM, 0.065 μg) and diluted cDNA 3 μL for the *GLO* reaction system and 6 μL for the *AG* reaction system. Owing to the already described low expression of *AG*, the volume of diluted cDNA was doubled for the *AG* reaction. Each reaction was amplified by two pairs of primers and processed with three biological replicates of the six floral organs extracted from multiple flowers: sepals, petals, petaloid staminodes, labellum, fertile stamens and carpels. *β-Actin* was amplified from the six floral organs as a reference. The real-time RT-PCR was run on an ABI7500 (USA) for 45 cycles, and cycling conditions were set as follows: pre-heating at 95 °C for 30 s, PCR reaction at 95 °C for 3 s followed by 60 °C for 30 s, and melt curve stage at 95 °C for 15 s, 60 °C for 60 s and 95 °C for 15 s. The relative quantization approach through a DeltaDelta CT method was used to analyse the results of real-time PCR experiments. ABI 7500 software v2.0.1 was used to collect data and carry out the statistical analysis.

## Results

### Isolation and sequence analyses of the two genes

Two positive sequences of MADS-box genes were obtained and identified as B- and C-class MADS-box homologue genes. The cDNA clones were named *CiGLO* and *CiAG*, respectively. Both cDNA clones encode MIKC-type proteins.

**Figure 2.**
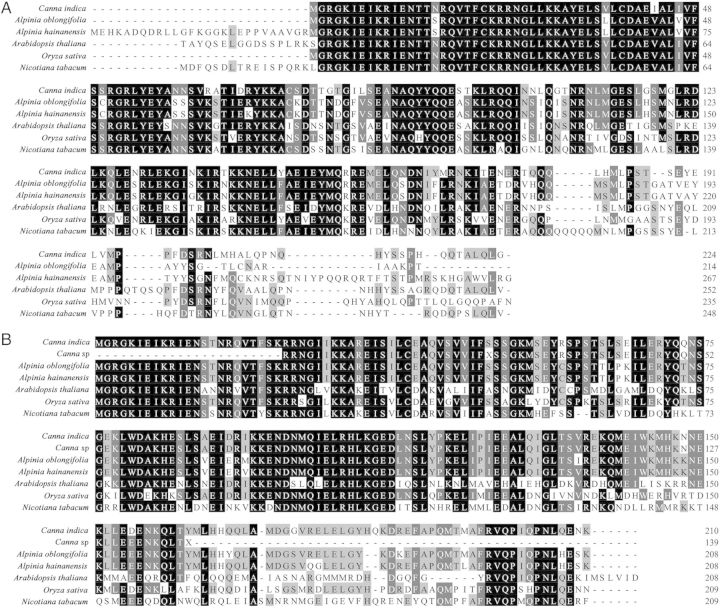
Alignments of deduced amino acid sequences. Identical amino acids with a consensus sequence are shaded, and a hyphen represents a gap inserted to optimize alignment. (A) *CiAG* and other proteins of the AG-like subfamily. (B) *CiGLO* and other proteins of the GLOBOSA subfamily.

*CiAG* cDNA has a length of 960 bp containing a poly-A signal encoding a protein of 224 amino acids with an open reading frame of 675 bp. The *CiAG* sequence is similar to the AG-like family in comparison to other MADS-box genes in GenBank including *A. oblongifolia* (ABB92624), *A. hainanensis* (AAT99428), *A. thaliana* (NP_567569), *O. sativa (*ACY26070) and *N. tabacum (*Q43585), and it is most similar to *AoAG* (DQ286724) from *A. oblongifolia* (Gao *et al.* 2006) and *AhMADS6* (AY621155) from *A. hainanensis* (Song *et al.* 2010) (Fig. 2A).

*CiGLO* cDNA is 870 bp long containing a poly-A signal encoding a protein of 210 amino acids, and the coding region is 633 bp long. *CiGLO* is similar in sequence to the GLOBOSA subfamily in comparison to other MADS-box genes from GenBank including *Canna* sp*.* (GU594945), *A. oblongifolia* (ABB92623), *A. hainanensis* (AAT99429), *A. thaliana* (NP_197524), *O. sativa (*NP_001045012) and *N. tabacum (*Q03416). The *CiGLO* gene shows strong homology with members in the GLO-like subfamily, and with 99 % similarity to *CsGLO2* (GU594945) of *Canna* sp. (a species located in America) (Bartlett and Specht 2010) as illustrated in Fig. 2B. *CiGLO* also has no less than 90 % similarity to *PoGLO2* (GU594953) of *Phrynium oliganthum*, *HwGLO2* (GU594953) of *Heliconia wagneriana*, *SjGLO2* (GU594935) of *Stromanthe jacquinii*, *OmGLO2* (GU594936) of *Orchidantha maxillarioides* as well as *MlGLO2* (GU594949.1) of *Marantochloa leucantha* as reported by Bartlett and Specht (2010).

### Phylogenetic analyses

**Figure 3.**
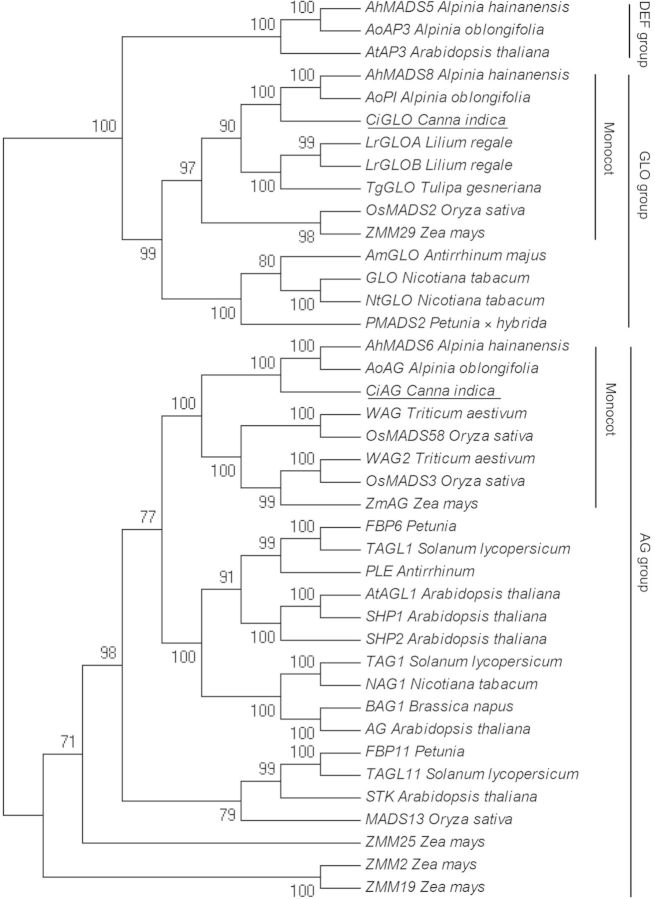
Phylogenetic tree constructed using maximum likelihood methods in MEGA. Numbers above the internal branches give bootstrap probabilities of >50. Genes of *C. indica* isolated in this study are underlined. Groups are indicated by the vertical bars at the right margin.

Phylogenetic analyses (Fig. 3) indicate that our recovered sequences fall into two distinct clades. Three groups can be distinguished by representative genes of the two clades, which are the AGAMOUS (AG), GLOBOSA (GLO) and DEFICIENS (DEF) subfamilies, respectively. Among the three groups, the two B-class subfamilies, DEF and GLO groups, were resolved as sister lineages and together form a sister clade to the C-class subfamily, the AG group. *CiAG* and *CiGLO* reported in this study are present in the AG and GLO clades, respectively. *CiAG* clustered together with *AhMADS6*, *AoAG* and other monocot C-class members and formed a monocot C sub-group with a 100 % bootstrap value. *CiGLO* clustered together with *AhMADS8*, *AoPI* and other monocot B-class members and formed a monocot B sub-group on a 97 % bootstrap value. D-class genes *SHP1*, *SHP2*, *FBP11*, *STK* and *MADS13* are included in the AG group and show close relationships with C-class members.

### Expression patterns of two MADS-box genes

**Figure 4.**
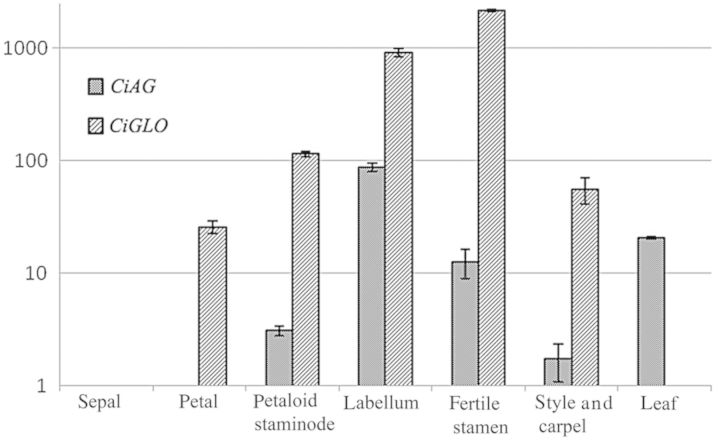
Expression patterns of *CiAG* and *CiGLO* in different floral organs and leaves of *C. indica*. The bars on top of each lane in the histogram mean standard error (SE).

The expression patterns of *CiAG* and *CiGLO* in different floral organs were analysed using real-time RT-PCR, as shown in Fig. 4.

*CiAG* expression signals were observed in both the outer and inner whorls of staminodes (including petaloid staminodes and the labellum), the fertile stamen and carpels. However, *CiAG* expression was not observed in sepals and petals as previously reported (Almeida *et al.* 2013). In other words, *CiAG* was expressed in floral organs of whorls 3 and 4, and not expressed in whorls 1 and 2, similar to the expression pattern of other AG-like homologues' based on the BC model. The strongest expression signal of *CiAG* was detected in the labellum of whorl 3o.

*CiGLO* expression was observed in petals, the outer whorl of petaloid staminodes, the labellum and the fertile stamen as well as carpels, while it was not observed in sepals. In other words, *CiGLO* was expressed in the floral organs of whorls 2, 3 and 4, but was not expressed in whorl 1. The strongest expression signal of *CiGLO* was detected in the inner whorl where the single fertile stamen is initiated.

In addition, the *CiAG* expression signal was also detected in the leaf, whereas no *CiGLO* expression signal was detected in the leaf.

## Discussion

Morphological and anatomical studies of *C. indica* (Kirchoff 1983) have shown that the sequence of floral organ initiation is as follows: calyx (sepals), corolla (petals), inner androecial whorl, outer androecial whorl and gynoecium. The developmental work of Kirchoff (1983) supports the hypothesis proposed by Eichler (1875), that the stamen, the labellum and one of the staminodes (the inner staminode) represent the inner androecial whorl while the remaining one or two petaloid staminodes represent the outer androecial whorl. The nature of the labellum was not formulated by Kirchoff (1983).

The most extraordinary and ornamental parts in the flower of *Canna* L. are staminodes, which are usually taxonomically interpreted as petals by people (Wu and Chen 1981, 2000). In the flora of China, *Canna* flowers are interpreted as having two whorls of petaloid staminodes, with the reflexed staminode in the inner whorl referred to as the ‘labellum’. The labellum is narrower than the outer whorl of staminodes and is useful in distinguishing among species of *Canna*. In the light of their statements (Wu and Chen 1981, 2000), the androecium members, except for the fertile stamen, were replaced by petaloid staminodes and the labellum. However, no detailed evidence has been put forward.

In the present study, two genes, *CiGLO* and *CiAG*, have been isolated from *C. indica*. Sequence characterization and phylogenetic relationships indicate that they belong to B- and C-class gene families, respectively. The described expression patterns in eudicot model species indicate that stamens are determined by B- and C-class genes in whorl 3, whereas C-class genes are essential for carpels in whorl 4 (Theißen 2001). The expression pattern in different floral organs analysed by qPCR shows that *CiAG* was expressed in whorl 3o of petaloid staminodes and the labellum, whorl 3i of petaloid staminodes plus the fertile stamen, and whorl 4 of carpels, while it was not expressed in whorl 1 of sepals and whorl 2 of petals. The qPCR results of *CiAG* are largely consistent with the expression patterns of C-class genes, as described in eudicot model species. In the study of Almeida *et al.* (2013), two copies of *AGAMOUS* genes were isolated from *C. indica*, and RT-PCR was used to assess the expression pattern. The genes *AG-1* and *AG-2* show an expanded pattern of expression when compared with the canonical BC model. *AG-1* is expressed in a gradient increasing from sepals to gynoecium while *AG-2* is evenly expressed in all floral parts studied except in petals. At that time, Almeida *et al.* (2013) did not amplify the full-length sequences for the two genes. According to the accession number, *AG-1* may be a part of *CiAG* of our study. The expression patterns of *AG-1* and *CiAG* are slighly different, which may result from the different reseach methods used. Moreover, the expression signal of *CiAG* has been detected in the leaf. *CiAG* expressed in non-floral tissue as well as in floral organs is in accordance with the research of Kim *et al.* (2005) in some basal angiosperms and of Chanderbali *et al.* (2006) in *Pe.am.AG* of *Persea* from the Lauraceae family. In this study, the authors proposed that the expression of *AG* in vegetative tissue is an evolutionary novelty (Chanderbali *et al.* 2006). Chanderbali *et al.* (2006) also hypothesized that the tepals of *Persea* and perhaps in other Lauraceae are derived from stamen primordia on the basis of gene expression and the occasional presence of tepaloid organs in stamen whorls.

*CiGLO* was expressed in whorl 2 of petals, whorl 3o of petaloid staminodes and the labellum, whorl 3i of petaloid staminodes and the single fertile stamen, and whorl 4 of carpels, while it was not expressed in whorl 1 of sepals. The expression of *CiGLO* in whorls 2, 3 and 4 seems consistent neither with the aforementioned modified monocot BC model (Van Tunen AJ 1993; Kanno *et al.* 2003; Kanno *et al.* 2007) nor with the shifting/sliding boundary model (Bowman 1997; Kramer *et al.* 2003), although it is still consistent with the canonical BC model of eudicots. The ‘shifting boundary’ (Bowman *et al.* 1991) and ‘sliding boundary’ (Kramer *et al.* 2003) models allow the boundary of expression of the B-class genes to ‘slide’ from that observed in *Arabidopsis* and *Antirrhinum* to one that includes the outer perianth whorl (outer tepals) of *Ranunculus*, *Tulipa* and other species with an entirely petaloid perianth. The extension of B-class gene expression into whorl 4 of carpels might explain the petaloidy observed in the style of *C*. *indica* (Glinos and Cocucci 2011).

Two copies of *GLOBOSA* were studied by Almeida *et al.* (2013), and both *GLO-1* and *GLO-2* show an expansion in their expression domains. *GLO-1* and *GLO-2* show great similarity to *CiGLO* of our study, but they are only partial CDS, which makes it difficult to address the relationship between them and *CiGLO*. Further data are required to see whether *CiGLO* is a new copy due to its divergent expression patterns. Broad expression patterns observed for *ZinGLO1*, *ZinGLO2*, *ZinGLO3* and *ZinGLO4* support a combination hypothesis which supports the idea that labellum identity is a result of the combination of multiple *GLO* homologues rather than expression of a single orthologue (Bartlett and Specht 2010). Whether the combination hypothesis is applicable in *Canna* needs broader investigation.

In the canonical BC model, simultaneous expression of B- and C-class MADS-box genes would result in the specification of stamen identity. *CiAG* and *CiGLO* are expressed simultaneously in the labellum, petaloid staminodes and the fertile stamen in the present study. If molecular evidence is sufficient to determine organ identity, one could claim that these results suggest that the three floral organs are of the same identity. However, despite their similar expression patterns, these organs present very different morphologies. The role of B- and C-class genes in determining final organ morphology awaits further studies. Other studies, such as stage-specific *in situ* hybridization, would be necessary for a more complete picture of the expression profiles during development. *In situ* hybridization experiments carried out on a C-class gene *AhMADS6* and two B-class genes *AhMADS5* and *AhMADS8* of *A. hainanensis* (Song *et al.* 2010) have shown a case similar to the present study, which has corroborated the hypothesis that the labellum of *A. hainanensis* originated from stamens. Recently, Miao *et al.* (2014) showed that the vascular bundle of the labellum of *C. indica* originates from two stamen primordia at an early developmental stage, providing anatomical evidence for the interpretation that the labellum should be regarded as a derivative of staminodes in the inner whorl (Miao *et al.* 2014). Although the molecular data presented in this study do not provide sufficient evidence to support that anatomical result, they are also not inconsistent with this interpretation. In accordance with the expression pattern of B- and C-class genes described by Almeida *et al.* (2013), our present data do not show differential expression patterns between fertile and sterile elements within the androecial whorl, and neither set of results can explain the observed petaloidy or how, mechanistically, the staminodes and the fertile stamen show distinct morphologies.

## Conclusions

Based on the expression patterns of B- and C-class genes, the molecular data presented in this study suggest that petaloid staminodes and the labellum are of androecial identity, in concordance with their position in the floral meristem and in agreement with largely described expression patterns in model species. However, these expression patterns are not sufficient to explain the distinct morphologies observed in staminodes and the fertile stamen. Broader and in-depth exploration of other copies of *GLO* and *AG* genes and sequence evolution, as well as a broader understanding of the involvement of B- and C-class genes in floral organ identity regulatory networks are required for a better understanding of the relationship between B- and C-class gene expression patterns and the floral organ morphology in *Canna*.

## Sources of Funding

Funding was provided by the Joint Fund South China Botanical Garden-Shanghai Institute of Plant Physiology & Ecology, National Natural Science Foundation of China (31200176, 31200246, 30900089) and Key Laboratory of Plant Resources Conservation and Sustainable Utilization, SCBG, CAS.

## Contributions by the Authors

Isolation of the sequences and real-time PCR analyses and data collection were accomplished by Q.F. following supervision by J.P.L. and the laboratory assistance of H.F.L. and P.Z. Phylogenetic analysis was performed by Y.F.K. Writing and editing and comprehensive revision of the manuscript was performed by Y.F.K. and A.M.R.A.

## Conflicts of Interest Statement

None declared.

## Accession Numbers

Novel sequences have been submitted to NCBI (http://www.ncbi.nlm.nih.gov). Accession numbers are *Canna indica AGAMOUS*, JQ180191 and *Canna indica GLOBOSA*, JQ180192.
